# On-substrate Enzymatic Reaction to Determine Acetylcholinesterase Activity in Whole Blood by Paper Spray Mass Spectrometry

**DOI:** 10.1007/s13361-018-2072-1

**Published:** 2018-10-02

**Authors:** Daniel O. Carmany, Phillip M. Mach, Gabrielle M. Rizzo, Elizabeth S. Dhummakupt, Ethan M. McBride, Jennifer W. Sekowski, Bernard Benton, Paul S. Demond, Michael W. Busch, Trevor Glaros

**Affiliations:** 1 Excet, Inc., 6225 Brandon Ave, Suite 360, Springfield, VA 22150 USA; 2 Biosciences Division, BioDefense Branch, US Army Edgewood Chemical Biological Center, Aberdeen Proving Ground, MD 21010 USA; 3 Toxicology and Obscurants Division, Analytical Toxicology Branch, US Army Edgewood Chemical Biological Center, Aberdeen Proving Ground, MD 21010 USA

**Keywords:** Acetylcholinesterase assay, Whole blood, VX, Paper spray

## Abstract

Currently, all assays measuring acetylcholinesterase (AChE) activity following a suspected nerve agent exposure leverage methodologies that fail to identify the agent. This limits the overall effectiveness and ability to administer proper countermeasures. As such, there is an urgent need to identify novel, rapid, and more comprehensive approaches to establish AChE activity, including identification of the toxicant. Paper spray mass spectrometry was used to monitor the activity of acetylcholinesterase, both in-solution and on modified hydrophobic paper surface. Hydrophobic paper surfaces were prepared using vaporized trichloro(3,3,3-trifluoropropyl)silane. In both approaches, mixtures of diluted human whole blood with and without VX were mixed with a non-endogenous AChE specific substrate, 1,1-dimethyl-4-acetylthiomethylpiperidinium (MATP+). Formation of the cleaved MATP+ product was monitored over time and compared to MATP+ to determine relative AChE activity. This on-substrate assay was effective at determining AChE activity and identifying the toxicant; however, determination of AChE activity in-solution proceeded at a slower rate. The on-substrate assay serves as a pioneering example of an enzymatic reaction occurring on the surface of a paper spray ionization ticket. This work broadens the range of applications relating to paper spray ionization-based clinical diagnostic assays.

**Electronic supplementary material:**

The online version of this article (10.1007/s13361-018-2072-1) contains supplementary material, which is available to authorized users.

**Graphical Abstract:**
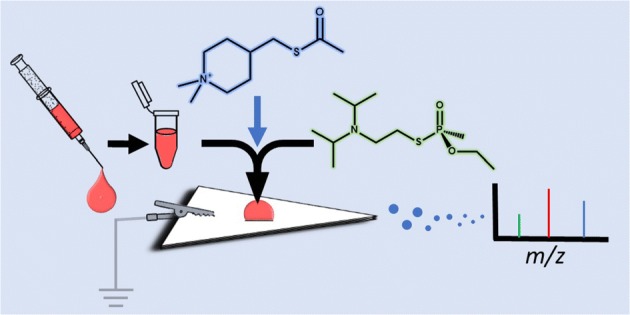
ᅟ

## Introduction

Acetylcholine (ACh) is the most widespread and best understood neurotransmitter. Research dating back more than 100 years suggested that an esterase is involved in its inactivation and removal from circulation. In 1932, Stedman et al. prepared the first extract of cholinesterase (ChE) and demonstrated its ability to breakdown ACh, to its choline product [1]. It is widely accepted that ChEs fall into two broad categories based upon substrate preferences, acetylcholinesterase (AChE) and butyrylcholinesterease (BChE) [2]. The primary role of AChE is to catalyze the hydrolysis of ACh in the cholinergic synapses within the central nervous system, the peripheral nervous system, and at neuromuscular junctions. When this function is impeded by pxcopy /y "\\pdgts1174\springer\jwf\template\Standard\\Contentchecker\*.dll" "C:\Program Files\Arbortext\APP-D Unicode\"xcopy /y "\\pdgts1174\springer\jwf\template\Standard\\Contentchecker\*.pyd" "C:\Program Files\Arbortext\APP-D Unicode\"xcopy /y "\\pdgts1174\springer\jwf\template\Standard\\Contentchecker\*.txt" "C:\Program Files\Arbortext\APP-D Unicode\"copy "\\pdgts1174\springer\jwf\template\Standard\\Contentchecker\*.dtd" "C:\Program Files\Arbortext\APP-D Unicode\"copy "\\pdgts1174\springer\jwf\template\Standard\\Contentchecker\*.jar" "C:\Program Files\Arbortext\APP-D Unicode\"copy "\\pdgts1174\springer\jwf\template\Standard\\Contentchecker\Validation*.*" "C:\Program Files\Arbortext\APP-D Unicode\"del "D:/Programs/ProductionJournal/Temp/ccc.bat"otent inhibitors, such as organophosphate (OP) pesticides, this leads to nerve signal transmission dysfunction. Like many proteins, AChE exists in several different isoforms which are widely distributed throughout the body; however, each isoform has identical catalytic properties [3]. Of these isoforms, the erythrocytic isoform (AChE-E) is the most accessible for assay development due to its location on red blood cells [4]. BChE is most abundant in the liver but is also readily found within blood fluids, commonly known as plasma cholinesterase. Although BChE hydrolyzes ACh, it is thought to function primarily as a scavenging enzyme for detoxification [5, 6]. It has also been implicated in a number of neurodegenerative and cardiovascular diseases [7, 8].

The primary clinical reason for measuring AChE or BChE activity is to diagnosis human exposure to pesticides, such as organophosphates (OPs) and carbamates, which act as cholinesterase inhibitors. To date, more than 200 different OPs and carbamates have been formulated into numerous products - commercial or otherwise [9, 10]. Given the widespread usage of these poisons and the relatively poor health surveillance, it is difficult to estimate the actual global health impact. Nonetheless, it is estimated that more than 3 million intentional poisonings occur globally each year resulting in roughly 200,000 deaths [11, 12]. Signs and symptoms from poisoning can vary greatly depending upon the compound, the dose, and length of exposure. Acute life-threatening exposures typically impact the respiratory centers within the brain stem and frequently result in fatalities [13]. Intermediate symptoms can also develop and are characterized by muscle weakness, especially in respiratory, neck, and limb muscle groups [14, 15]. These can occur hours, days, or weeks post-exposure. Diagnosis of ChE-inhibiting compounds can be difficult, especially since the compounds can have varying degrees of affinity for AChE and BChE. Additionally, some of the OPs, especially chemical warfare agents (CWAs), can bind irreversibly [16, 17]. For acute exposure diagnosis, ChE test data is typically only ordered in instances where the poison is known or suspected to be a ChE inhibitor. Conversely, ChE inhibition is quite useful for medical surveillance applications, especially for employees who are at higher risks of exposure in the workplace [4].

Although several different ChE inhibition assays have been developed since the 1940s, the delta-pH assay and the Ellman assay are the most commonly utilized [18, 19]. These assays are based upon the products which are created when AChE/BChE cleaves its target substrate. In order to establish the level of inhibition of both enzymes, two separate assays must be performed, typically with the inclusion of a BChE-specific inhibitor while measuring AChE activity. The delta-pH technique is the mostly widely used, as it is the current routine test used to monitor more than 15,000 people annually at the U.S. Army Center for Health Promotion and Preventive Medicine (USACHPPM) [4, 20]. For this test, acetylcholine is the preferred substrate, which, when cleaved, produces acetic acid and choline. The production of acetic acid is monitored via observing the pH change over time. Even though this technique is well-established, it requires 17 minutes to perform the assay, which severely limits the throughput and feasibility of implementation. The more sensitive Ellman assay is a photometric-based system that uses acetylthiocholine as the substrate. When cleaved, yielding thiocholine, it reacts with 5,5′-dithiobis-2-nitrobenzoic acid to produce a colorimetric response which is measured with a spectrophotometer and uses only 10 μL of blood.

Paper spray mass spectrometry (PS-MS) is an ambient ionization technique that has been used to detect and quantitate a wide range of small molecules from complex matrices [21]. This approach uses small amounts of solvent and can be performed in as little as one minute. Several applications have been published utilizing this technique, including analysis of pesticides [22, 23], fungicides [24, 25], illicit drugs [26, 27], bacterial identification [28], and direct aerosol capture and identification [29]. Modification of the paper surface with compounds such as carbon nanotubes [30], metal-organic frameworks (MOFs) [31, 32], and silanes [33] has also been utilized to target specific applications. Yan *et al*. demonstrated the advantages that paper spray has in measuring enzymatic activity, by bypassing the standard convention requiring a multi-step derivatization process of reaction products by directly measuring the formed products [34]. Their assay measuring aminotransferase activity was carried out in two steps in micro-centrifuge tubes and spotted onto the paper spray substrate for analysis.

In this work, a novel paper spray-based assay was developed which measures relative acetylcholinesterase activity and is capable of identifying the intoxicating nerve agent, VX (Ethyl ({2-[bis(propan-2-yl)amino]ethyl}sulfanyl) (methyl) phosphonate). The feasibility of this was first tested by performing the reactions in-solution and analyzing by PS-MS. The study involved the use of a AChE-specific substrate known as MATP+ (1,1-dimethyl-4-acetylthiomethylpiperidinium iodide). To leverage the full potential of the paper spray technique and improve the assay, an analysis was also performed directly on the spray substrate. As the application of PS-MS continues into the clinical space for detection of biomolecules, the incorporation of an on-substrate enzymatic step will become increasingly important.

## Experimental

### Caution

Due to the acute hazards with VX, all experiments involving VX were performed by qualified personnel in certified chemical fume hoods equipped with an advanced filtration system that protects the user and the environment at the US Army’s Edgewood Chemical Biological Center (Edgewood, MD) according to all Federal, State, and International guidelines.

### Reagents

All materials were purchased from Sigma Aldrich (St. Louis, MO, USA) unless otherwise noted. Human blood was purchased from Innovative Research (Novi, MI, USA). Methanol Optima grade was obtained from Fisher Scientific (Waltham, Massachusetts, USA). Paper spray cartridges were purchased from Prosolia Inc. (Indianapolis, IN, USA) and modified in-house as described previously [33]. Briefly, Prosolia cartridges were carefully disassembled. The Whatman paper portion of the cartridges were placed onto a rack in a desiccation chamber with 100 μL of trifluoropropyl trichlorosilane, and the chamber was placed under vacuum. The vacuum caused the trifluoropropyl trichlorosilane to vaporize. The Whatman paper was exposed to the vapor for 4 h. After the exposure, the chamber was opened and the cartridges were reassembled.

### Assay Development

**Figure 1:**
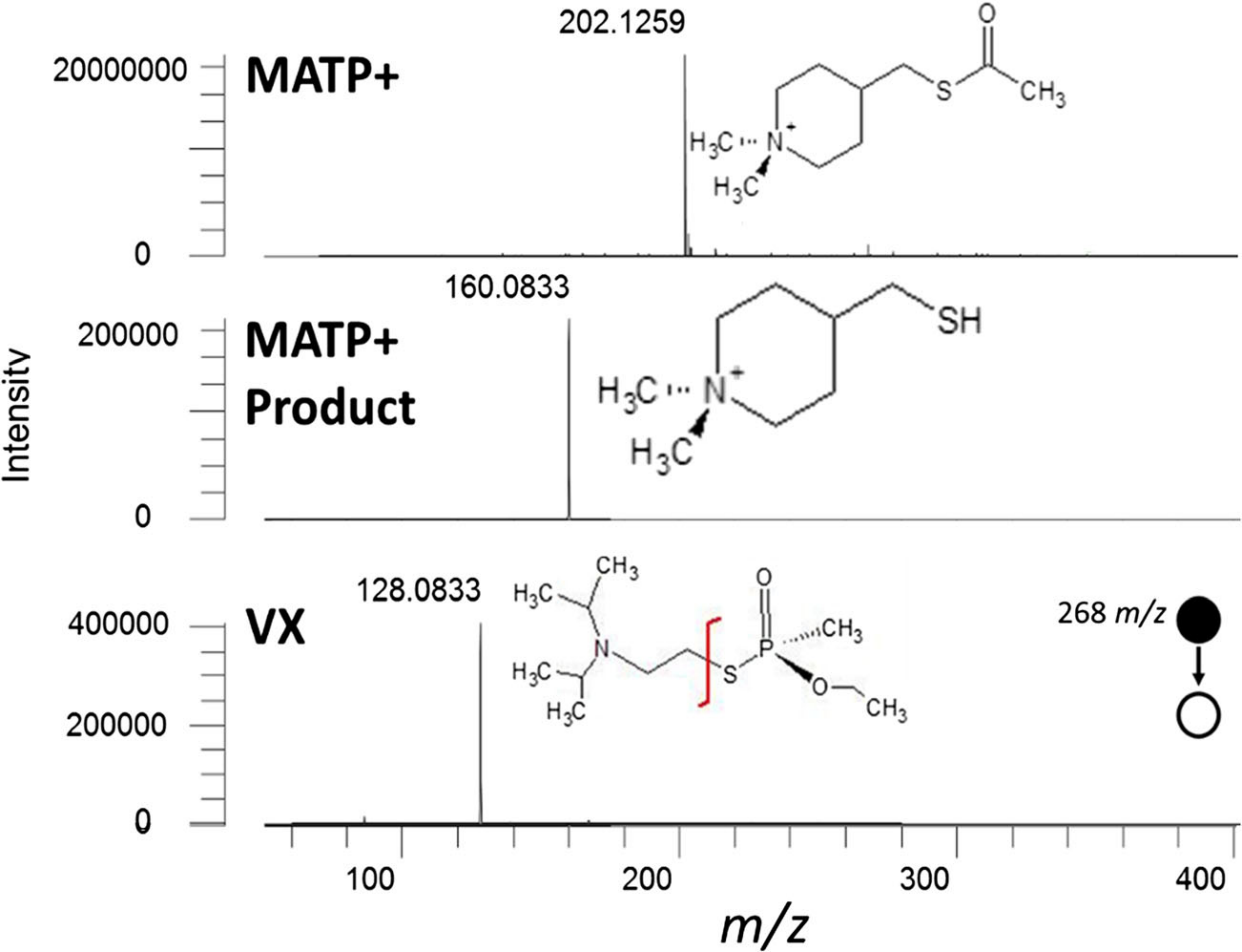
Detection of MATP+, MATP+ product, and VX via PS-MS. Not only is the activity of the AChE detectable, but also the source of inhibition, VX

Mass analysis was performed using a Thermo Fisher Orbitrap Elite mass spectrometer (Thermo Scientific Inc., San Jose, CA, USA). To establish target compounds’ accurate masses, transitions, and optimal collision energies, each compound was directly infused and analyzed by electrospray ionization. Pertinent settings for MS analysis are as follows: capillary temperature: 325 °C, s-lens = 60% RF level, ionization voltage = 5.0 kV. For MATP+ high resolution accurate mass spectrometry (HRAM) was used following *m*/*z* 202.0836 (Fig. 1). VX data was acquired using collision induced dissociation (CID) tandem mass spectrometry (MS/MS) following *m*/*z* 268.1494 → 128.0833 with a fixed collision energy (CE) of 30 V. The MATP+ product fragment was identified and optimized by incubating *Electrophorus electricus* (electric eel) AChE with the MATP+ substrate. Briefly, eel AChE was diluted to 1 μg/mL (*m*/*v*) in 10 μg/mL of MATP+ (*m*/*v*) and incubated for 1 h at 37 °C with agitation. The reaction on the cartridge was then directly infused into the mass spectrometer to establish accurate mass for subsequent analysis. The MATP+ cleavage product was also analyzed by HRAM resulting in an *m*/*z* of 160.0837.

### Human Blood Spiked with VX

A 90 μL aliquot of whole human blood was mixed with 10 μL of VX diluted in MeOH at various concentrations. The mixture was agitated on a shaker block at 37 °C for 30 min and allowed to cool to room temperature. VX was obtained in-house, in accordance with international accords, and evaluted for purity via NMR.

### Paper Spray Mass Spectrometry

A custom–built paper spray ionization housing fitted to a Thermo Fisher Nanospray Flex Ionization source was used in this study, along with the PS cartridges available from Prosolia, Inc. Sample was applied to the spray substrate depending upon the reaction method detailed below. For all experiments the spray solvent was 95:5 methanol/water (*v*/*v*) with 0.01% formic acid (*v*/*v*). A total of 80 μL of spray solvent was aliquoted onto the PS substrate—approximately 10 μL at the front of the substrate and 70 μL at the rear. Mass analysis for the paper spray samples was performed using a Thermo Fisher Scientific Orbitrap Elite as described previously in the method development section with some changes. Briefly, the MS method run time was 1.3 min, broken down into 3 time segments with varying spray voltages: 0–1.0 min, +5 kV; 1.0–1.15 min, 0 kV; and 1.15–1.30 min, −4.5 kV. The mass resolution was 15,000.

### In-Solution AChE Activity Assay

Human blood was diluted 1:100 in 50 μg/mL MATP+ (*m*/*v*). The reaction was placed into a 37 °C shaking heater block with 10 μL aliquots removed at 5 min intervals for paper spray analysis. Relative AChE activity was determined by using the change in area ratio of MATP+ (*m*/*z* 202.0836)/MATP+ product (*m*/*z* 160.0833) at the various time points. Area of each ion was extracted from the chromatogram within a 10 part per million mass tolerance window from MS1 scans using XCalibur V2.2 SP1.48. As a positive control for inhibition, human blood samples spiked with a fixed concentration of VX were also assayed in parallel. The PS-MS based assay was compared to a standard Ellman assay for AChE inhibition as described previously [20].

### On-substrate AChE Activity Assay

The reaction was prepared as described in the in-solution protocol in the methods section above with and without VX. Ten microliters of the reaction mixture was placed onto a silanated paper cartridge treated by the above method. Data was processed as described above.

## Results and Discussion

### In-solution AChE Activity Assay

**Figure 2:**
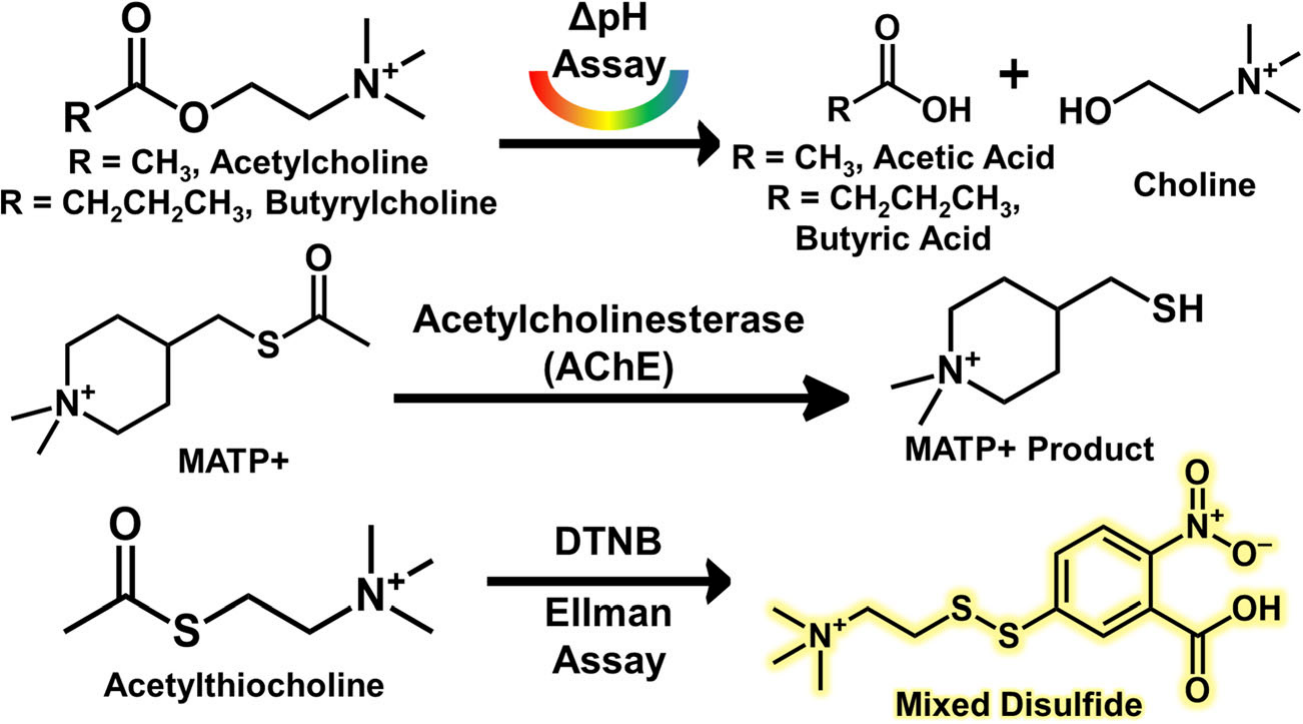
(Top) A traditional pH based detection of acetylcholinesterase assay with the products lowering the overall pH of the solution with the formation of the acid cleavage products. (Middle) The non-endogenous cleavable MATP+ molecule used as an internal standard of the newly developed assay. (Bottom) A traditional Ellman assay with reporter molecule detection via spectroscopy

**Figure 3:**
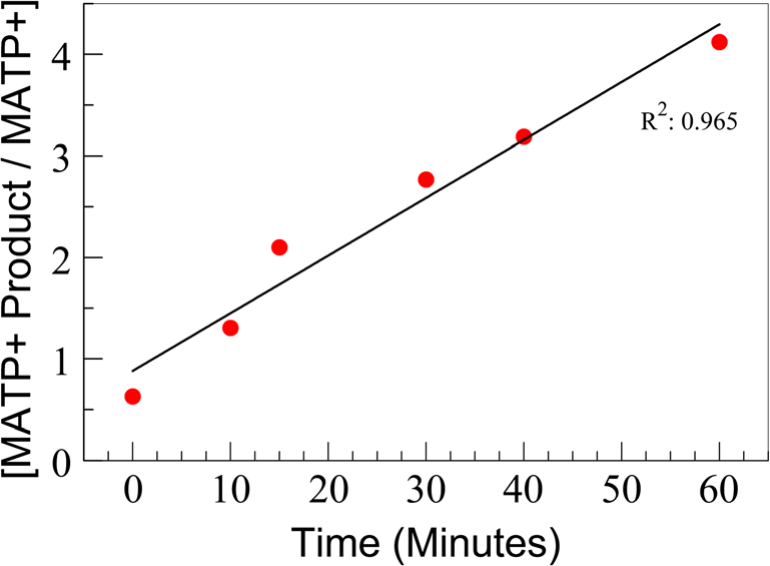
In-solution determination of the rate of MATP+ product formation over time utilizing *Electrophorus electricus* (electric eel) AChE. This plot is representative of three independent experiments (*N* = 3)

The delta pH and Ellman assays are detailed in Fig. 2, along with the PS-MS AChE substrate used here. The PS-MS AChE assay is simplistic; however, the method has the added benefit of being able to detect the toxicant *in situ*, while the other two assays cannot. MATP+ was selected for use as the AChE substrate due to a high specificity for the enzyme when compared to the BChE enzyme. As a proof of concept, electric eel AChE was used to perform a time course for analysis by PS-MS. The analysis established there were no confounding ions resulting from the paper itself, and there was clear time dependence for the formation of the product (Fig. 3). By comparing the conversion of the substrate to the product, it is possible to assess the enzyme’s relative activity without the need for an internal standard for normalization. This is consistent with the delta pH assay [19]. Presently the Ellman-based assay, which is fielded by the U.S. Armed Forces, requires normalization to heme due to the required dilution step of the sampled whole blood [20]. Since time dependence is critical for assay development, we first determined if a quenching step is needed. As shown in Fig. S1, once the assay is spotted on the paper, the reaction no longer persists as there was no increase in product formation compared to the in-solution control.

**Figure 4:**
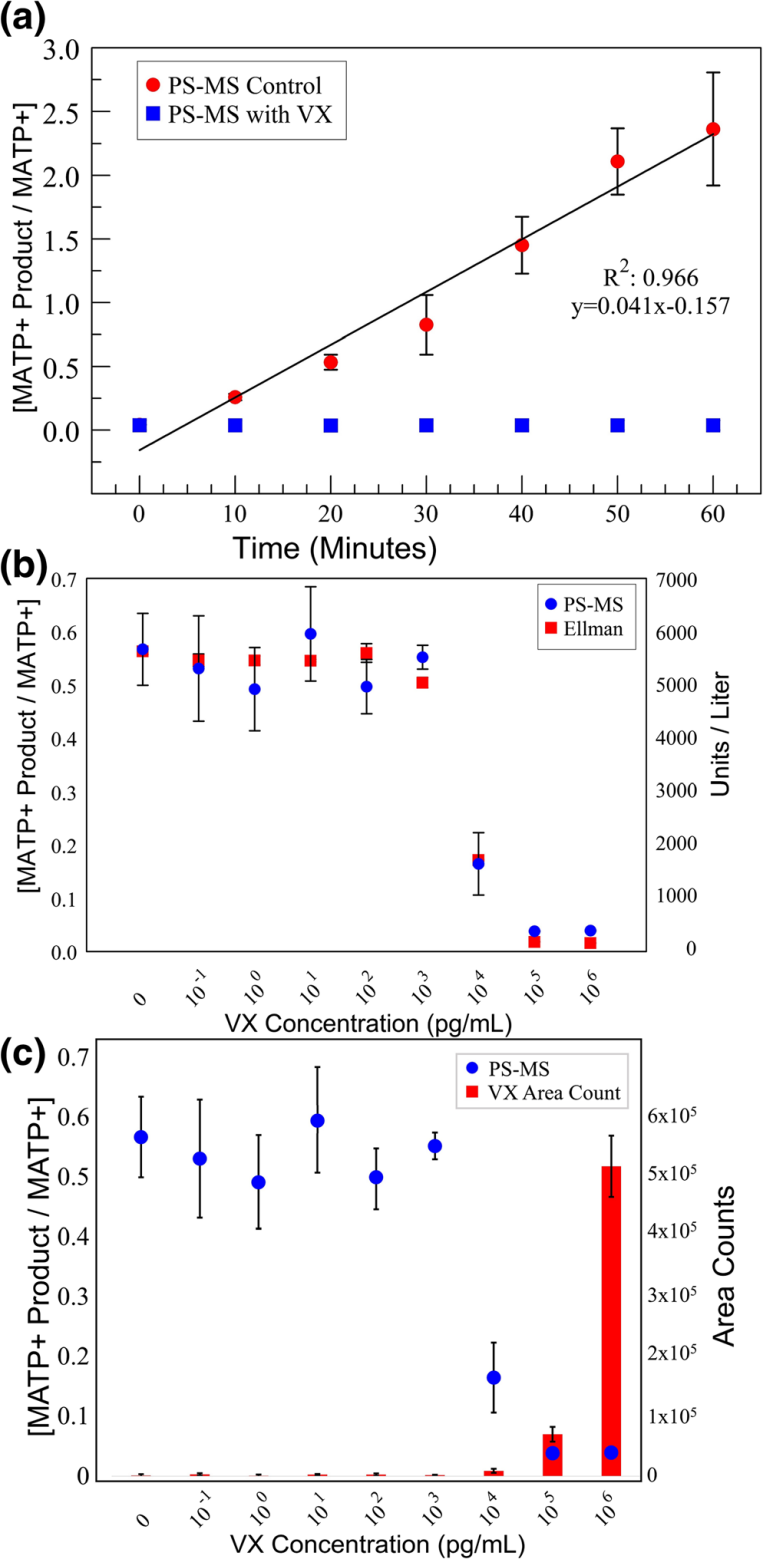
In-solution AChE assay analyzed by PS-MS. (**a**) 1% human blood was analyzed for AChE activity with (blue, squares) and without (red, circles) exposure to VX at a single concentration (0.5 μg/mL) while being incubated at 37 °C. (**b**) Comparison of the effectiveness of a paper spray-based assay to the traditional Ellman assay at eight different concentrations spanning seven orders of magnitude following 30 min of incubation at 37 °C. (**c**) 1% human blood was spiked with varying concentrations of VX (1 pg/mL → 1 μg/mL) and allowed to incubate for 30 min at 37 °C. The formation of MATP+ product decreased linearly until complete inhibition at ~ 10 ng/mL while increasing concentrations of VX were also detected

Initally, we optimized the ratio of blood to substrate by varying their respective concentrations with the goal of observing the maximal amount of product formation in 30 minutes without introducing excessive error. From these experiments, we determined that 1% blood diluted in water, resulted in the most reproducible data set. The dilution in water is a critical step as it results in the lysis of red blood cells, releasing AChE to perform the assay. Whole blood was diluted to 1% (*v*/*v*) with a fixed 10 μg/mL concentration of MATP+ in a centrifuge tube, followed by incubation at 37 °C for a time course with periodic analysis via the developed PS-MS technique. Since the nerve agent VX has been shown to directly inhibit AChE, it was used as a control for assay development [35, 36]. As shown in Fig. 4a, VX inhibited the formation of the product; whereas, the MATP+ product continued to form in a linear (*R*^2^ = 0.966) and time-dependent fashion throughout the duration of the 60 minute time course. When concentrations of VX were titrated in at increasing levels, starting at 0.1 pg/mL to 1 μg/mL, decreasing levels of AChE activity were observed (Fig. 4c). The linear portion of the curve covered seven logs of VX concentrations demonstrating sensitivity over a wide range of physiologically relevant concentrations, assuming that an 8-μg/kg dose is lethal via intravenous exposure for the average sized adult (82 kg) [36]. The PS-MS AChE assay was then compared to the traditional Ellman assay by spiking in seven different levels of VX (0.1 pg/mL to 1 μg/mL). At all concentrations, the two assays were in good agreement (Fig. 4b). In the PS-MS assay, VX was freely available for detection. Detecting unbound OP in whole blood obtained from victims of an actual chemical warfare event is unlikely unless there were lethal levels of exposure. As such, measuring AChE activity would be futile. However, our laboratory recently demonstrated it is possible to detect physiologically relevant OP hydrolysis products in blood rapidly using the paper spray technique [37].

### On-substrate AChE Activity Assay

**Figure 5:**
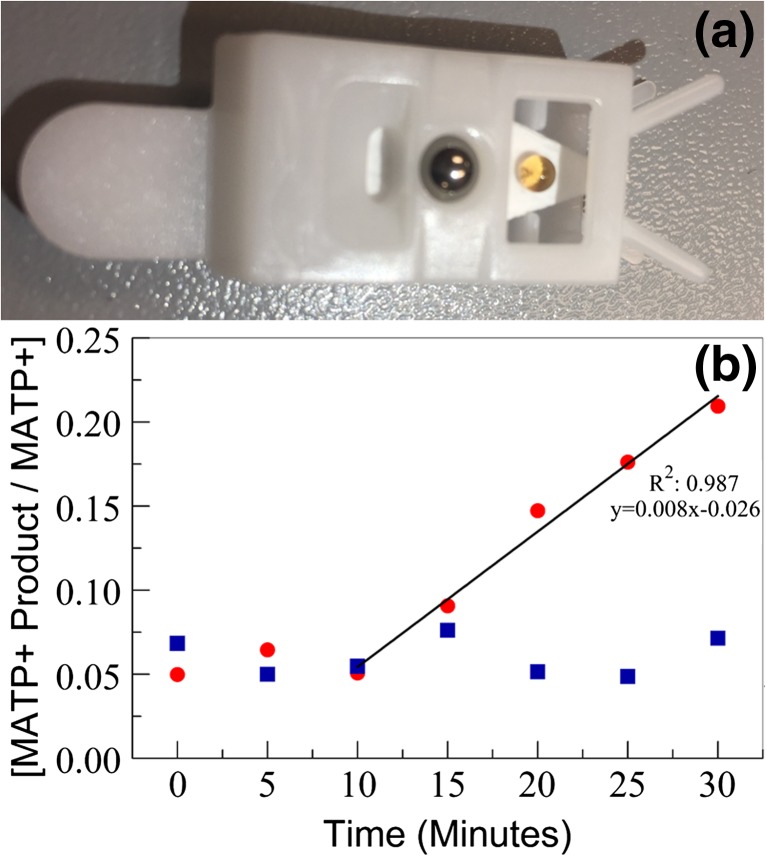
On-substrate AChE assay analyzed by PS-MS. The silane treatment caused the surface of the paper matrix to become hydrophobic. (**a**) To test the hydrophobicity 10 μL of 1% blood diluted in water was placed onto the cartridge, forming an aqueous sphere. (**b**) A mixture of 1% whole human blood with MTAP+ incubated with (blue square) and without (red circle) 0.5 μg/mL VX was prepared. 10 μL was placed on to several silanated cartridges and incubated at 37 °C. At 5 minute intervals, a ticket was removed from the incubator and analyzed by PS-MS for AChE activity. This plot is a representation of three independent experiments (*N* = 3)

To take advantage of paper spray’s strengths, speed and minimal sample prep, we attempted to perform the PS-MS AChE assay directly on the paper substrate, bypassing the need for incubation in a micro-centrifuge tube. Initially, the reaction on filter paper using dried blood spots was tried. These experiments were performed because there are multiple reports in the literature indicating that AChE enzymatic activity is retained in dried blood spots for up to at least 30 days [38, 39]. Several conditions were tested including varying the blood concentration, blood dryness, substrate concentrations, and incubation conditions. In all cases, the enzymatic reaction failed to generate a measurable amount of product. Based upon observations, we hypothesized that this malfunction was due to the MATP+ substrate failing to intercalate effectively into the blood spot. To concentrate the reaction components while keeping them in-solution, the paper surface was silanated to make it hydrophobic. This forced the reaction to stay in-solution as a droplet or sphere on the paper surface during the incubation period permitting the reaction to proceed (Fig. 5a). This methodology allowed the reaction to generate the product; however, the enzymatic reaction did have a 10 minute lag and proceeded at a slower rate, as indicated by the 80% decrease in slope during the linear portion of the curve (Fig. 5b). These observations were attributed to the absence of an effective way to mix the droplet during incubation. The on-substrate assay presented to the paper spray system could be leveraged to perform on-substrate enzymatic reactions with minimal sample handling for other clinical assays or applications.

## Conclusions

The work illustrates that PS-MS can not only be used to detect enzymatic products, but has the potential to significantly streamline enzymatic-based assays especially due to the rapid, simplistic PS process. Because the detection technique utilizes mass spectrometry without an additional reaction from the enzymatic product, the system has the potential to be a useful platform for a diverse array of assays. This is exemplified by the ability to not only assess the relative enzymatic activity but also identify the toxicant in situ. In the event the toxicant is not detectable, it would be possible to modify this approach to directly detect metabolites of suspected toxicants. Although we leveraged high resolution mass spectrometry in this work, the assay could easily be amenable to a low-resolution mass spectrometer. This is important as it makes it possible to perform these types of techniques on miniaturized portable mass spectrometers in the field or at a clinical site.

## Electronic supplementary material

ESM 1 (PDF 46.8 kb)
