# Supplementary material for: On-substrate Enzymatic Reaction to Determine Acetylcholinesterase Activity in Whole Blood by Paper Spray Mass Spectrometry
Source: J Am Soc Mass Spectrom. 2018 Oct 2;29(12):2436–42. doi: 10.1007/s13361-018-2072-1 (PMC6276064; doi:10.1007/s13361-018-2072-1)
Supplement: Supplementary file 1 — (PDF 46.8 kb) [file 13361_2018_2072_MOESM1_ESM.pdf]

Supplemental Material for:

On-Substrate Enzymatic Reaction to Determine Acetylcholinesterase Activity in Whole Blood by Paper Spray Mass Spectrometry

Daniel O. Carmany,[1] Phillip M. Mach,[2] Gabrielle M. Rizzo,[1] Elizabeth S. Dhummakupt,[2] Ethan M. McBride,[2] Jennifer W. Sekowski,[2] Bernard Benton,[3] Paul S. Demond,[1] Michael W. Busch,[1] Trevor Glaros[2*]

[1]Excet, Inc. 6225 Brandon Ave, Suite 360, Springfield, VA, 22150, USA

[2]Biosciences Division, BioDefense Branch, US Army Edgewood Chemical Biological Center, Aberdeen Proving Ground, MD, 21010, USA

[3]Toxicology and Obscurants Division, Analytical Toxicology Branch, US Army Edgewood Chemical Biological Center, Aberdeen Proving Ground, MD, 21010, USA

Corresponding author: trevor.g.glaros.civ@mail.mil

Abstract:

Currently all assays measuring acetylcholinesterase (AChE) activity following suspect nerve agent exposure leverage methodologies that fail to identify the agent. This limits the overall effectiveness and ability to administer proper countermeasures. As such, there is an urgent need to identify novel, rapid, and more comprehensive approaches to establish AChE activity, including identification of the toxicant. Paper spray mass spectrometry was used to monitor the activity of acetylcholinesterase, both in-solution and on a modified hydrophobic paper surface. Hydrophobic paper surfaces were prepared using vaporized trichloro(3,3,3-trifluoropropyl)silane. In both approaches, mixtures of diluted human whole blood with and without VX were mixed with a non-endogenous AChE specific substrate, 1,1-dimethyl-4-acetylthiomethylpiperidinium (MATP+). Formation of the cleaved MATP+ product was monitored over time and compared to MATP+ to determine relative AChE activity. This on-substrate assay was effective at determining AChE activity and identifying the toxicant, however determination of AChE activity in-solution proceeded at a slower rate. The on-substrate assay serves as a pioneering example of an enzymatic reaction occurring on the surface of a paper spray ionization ticket. This work broadens the range of applications relating to paper spray ionization-based clinical diagnostic assays.

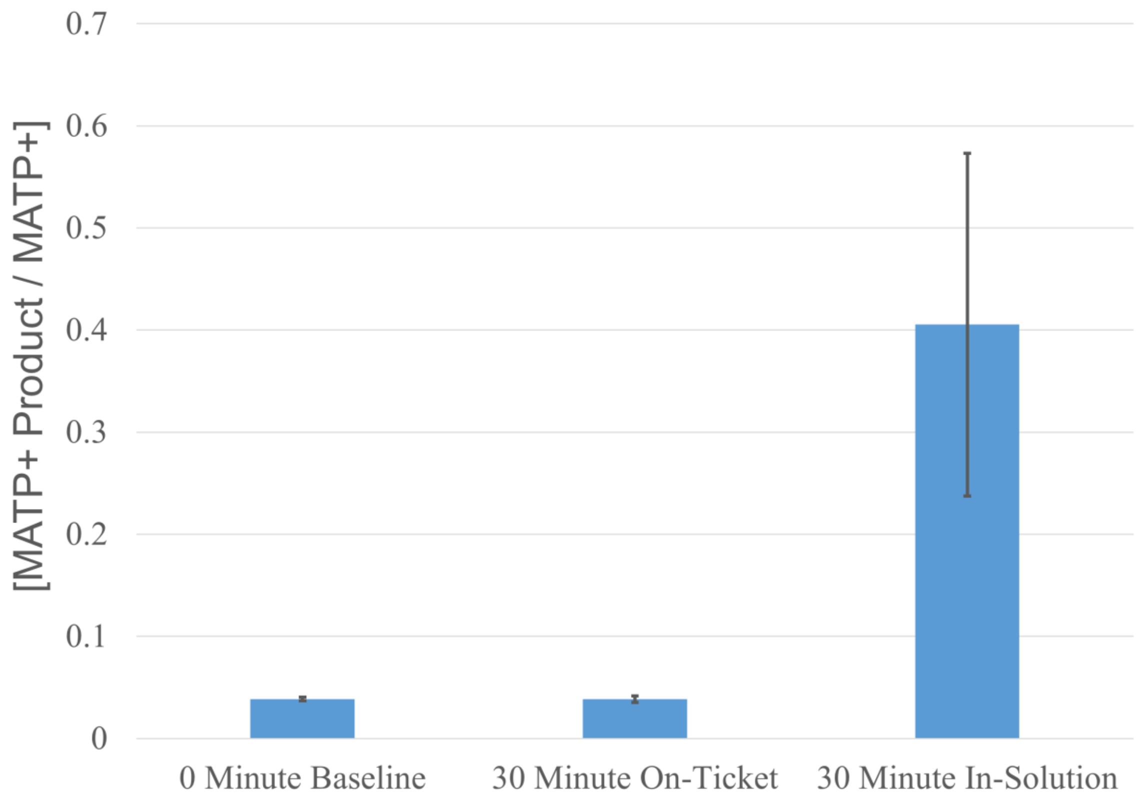

Figure S1. Response of AChE assay when applied directly to the untreated PS substrate at 0 and 30 minutes, with minimal generation of the cleavage product. Successful cleavage of MATP+ in solution at 30 minutes, showing activity when paper substrate is absent.
